# EFL Teachers' Optimism and Commitment and Their Contribution to Students' Academic Success

**DOI:** 10.3389/fpsyg.2021.752759

**Published:** 2021-10-18

**Authors:** Di Lu

**Affiliations:** Education School, Huaibei Normal University, Huaibei, China

**Keywords:** EFL teachers' optimism, students' academic success, academic success, teacher commitment, positive psychology

## Abstract

The present review study determines to scrutinize EFL teachers' optimism and commitment and their contribution to students' academic success. Academic optimism, as a new construct, is evolving from the examination of the positive psychology (PP), societal principal, and communal school assets that affect the attainment and success of all learners. In addition, within the past decades, commitment has received a great level of consideration, principally in the domain of structural research. The straightforward perseverance of this review is to extend the concept of academic optimism to individuals, that is, to hypothesize theoretical optimism and approve the efficacy of this paradigm at the instructor level in relation to students' academic success. According to the literature review, the definitions of these constructs, namely teachers' optimism and commitment, and students' academic success, as well as empirical studies in this domain are presented. As a conclusion, this study, to some extent, promotes the educators' mindfulness about their commitment. In this respect, pedagogical implications for teachers, school principals, teacher-trainers, and future researchers are presented, and new guidelines for further research are determined.

## Introduction

The success of learners will be consistently at the core of learning strategy and training, and nurturing learner accomplishment levels continues to be perhaps the most basic of objectives that instructors have had for quite a long time (James et al., 2001; Strong et al., 2001). Academic success is a journey that is not easily accomplished or ensured, and approaches to making scholastic progress may fluctuate (Alig-Mielcarek and Hoy, 2005; O'Donnell and White, 2005). Learners' success can be credited to an assortment of explanations; however, it is not obvious which components are liable for the transformation (Goddard et al., 2009). Success is accomplished when the three parts are available and completely created in a school situation; consequently, attempts are made to remove the socioeconomic obstacle to accomplishment and interpret it as less of an issue in the formula for learners' achievement (Hoy et al., 2006; McGuigan and Hoy, 2006).

Lately, positive psychology (PP) ideas like self-efficacy, emotional well-being, execution, anxiety, exhaustion, sadness, and nervousness have been a significant focal point of the study (Peterson, 2009; Meyers et al., 2013; Seear and Vella-Brodrick, 2013; MacIntyre and Mercer, 2014; Gabryś-Barker and Gałajda, 2016; MacIntyre et al., 2016; Dewaele et al., 2019; Mercer and Gregersen, 2020; Budzińsk and Majchrzak, 2021; Wang et al., 2021). As declared by Seligman (2000), PP investigates and clarifies ideal conditions. To Seligman (2002), PP plans to enhance personal satisfaction as opposed to tackling existing issues; similarly, Kurz (2006) expressed that PP centers around an appropriate concentration of skills and limits instead of issues. It is a total term that encompasses well-being, fulfillment, joy, demonstrative fulfillment, confidence, reliance, and zeal for work, all involving individual constructive encounters (Seligman and Csikszentmihalyi, 2000; Hoy and Tarter, 2011). Self-efficacy, in particular, is maintained to be a progressive and encouraging component that ought to be investigated in the academic study (Hoy and Tarter, 2011). Examining positive feelings (particularly optimism), characteristics, and establishments, positive psychologists recognize circumstances where people flourish and prosper. Such a climate is absolutely what most teachers want the class to be. A positive teaching space would accentuate the prospects and potentials, flexibility, and trust (Piliavin, 2003; Ryff and Singer, 2003; Wethington, 2003; Hoy et al., 2006). As stated by Pajares (2001), when taking a glance at the class setting, optimistic instructors center around the positive characteristics of learners, classes, schools, and networks. Optimism is the direct opposite of weakness, which is an approach to extend individual mechanisms (Seligman, 2006); it highlights hope, obligation, and an overall positive attitude to life.

The educational optimism has its hypothetical foundations in Bandura's social intellectual hypothesis, Coleman's community principal hypothesis, Seligman's academic optimism, and Hoy and his associates' research on the values and environment of the schools. This concept has been investigated as a discrete instructor trademark (Hoy et al., 2008; Beard et al., 2010) and as the assets of the faculty (Hoy et al., 2002; Hoy and Miskel, 2005; McGuigan and Hoy, 2006). Nonetheless, at the two points, the hypothetical reinforcements and their parts continue as before—the only contrast being that the item of scrutiny is either the discrete instructor or the department. Optimism gives an establishment to the paradigm and inspirational essence of scholastic optimism in that it elicits perspectives on instructors as skilled, learners as willing, guardians as caring, and the assignment as feasible (Hoy et al., 2006).

Optimism, in general, envisions positive outcomes (Carver et al., 2010). Besides, when optimistic people experience difficult yet conceivably conquerable impediments, they attempt to resolve the issues and emphasize their objectives, adapting to critical thinking, and cautious arrangements (Carver et al., 2010). Optimistic people are bound to show strength when confronting testing circumstances despite the fact that they may show moderate advancement (Snyder et al., 2002). Optimism assists learners with adapting to difficulties in school (Miranda and Cruz, 2020). Moreover, it has been shown that learners practice greater strength levels with more optimism levels (Dawson and Pooley, 2013). Optimistic people are stronger when confronting difficulties compared to less enthusiastic and confident people (Kleiman et al., 2017; Gómez-Molinero et al., 2018; Pathak and Lata, 2018).

Past and current research (Chang and Sanna, 2001; Diener et al., 2003; Makikangas and Kinnunen, 2003; Eid and Diener, 2004) have discovered that optimism altogether envisages a few parts of personal well-being. As stated by Shnek et al. (2001) and Vickers and Vogeltanz (2000), optimism is negatively identified with burdensome symptomatology both in general populaces as well as in populaces with different chronic conditions, like a cardiovascular infection. It is similarly a critical display of physical and spiritual working in people undergoing diverse ailments (Fournier et al., 2002). Optimism has been discovered to be related to operational issues and health both straightforwardly and in a roundabout way by means of, for instance, confidence (Taylor and Armor, 1996). Numerous inquiries have also conveyed a positive rapport between educational optimism and the learner and school success (McGuigan and Hoy, 2006; Smith and Hoy, 2007). Academic optimism was defined by Hoy et al. (2006) as a more contemporary construct than scholastic optimism. It considers theoretical self-efficacy, confidence, and educational implications not only at the individual but also at the administrative level (Hoy and Tarter, 2011). Educational optimism is described as a professor's reliance on paternities and learners about their educations, self-efficacy to pass through the connected difficulties, for the moment highlighting researchers supporting learners to flourish (Hoy et al., 2006).

In addition, commitment is a mental connection to an association wherein individuals give their faithfulness to its beliefs and objectives. Teacher commitment is the expressive security that instructors exhibit in the direction of their work. It has been perceived as quite possibly the most basic component in successful instructing. In this way, as stated by Altun (2017), educators with significant degrees of commitment can affect the education and accomplishments of their learners. Teacher commitment is related to establishing a powerful learning climate wherein learners upgrade their capacities for more prominent accomplishments, and it is a core power that pushes instructors to display improved job execution (Altun, 2017). The high loyalty of an educator to the school shows a high receptivity to class beliefs and an eagerness to apply exertion to school tasks and remain in school (Huang et al., 2016). Teacher commitment to instructing alludes to the degree to which educators are happy with their work and prospectively distinguish themselves as instructors (Park, 2005). Teacher commitment is connected to generating an operational learning situation in which learners improve their capabilities for superior and better accomplishments (Tsui and Cheng, 1999). Teacher commitment has been proposed as a basic component of the accomplishment of faculty instruction, which is related to educators' work performance, non-attendance, turnover, and disposition toward school and learners' scholarly accomplishments (Elliott and Crosswell, 2002). The are two main reasons to accentuate teacher commitment. First, it is an inner power coming from educators themselves who have a need for more noteworthy duties, variety, and challenges in their work as their instructive ranks are developing. Second, it is an outer power originating from the change program looking for exclusive requirements and responsibility, which are reliant upon teachers' wilful commitment.

Inquiries have guaranteed that teacher commitment is a basic indicator of an educator's work execution and the quality of teaching (Tsui and Cheng, 1999). Educators are relied upon to be committed to their work at all times, yet their commitment exclusively relies upon the foundation of the school, mentalities exhibited by their directors, school size and culture, and head initiative (Huang et al., 2016). The commitment arises when they show a more significant level of execution by taking extra responsibilities in their obligations (Sarikaya and Erdogan, 2016). Therefore, one might say that commitment is the capacity of instructors to truly embrace a school's long- and short-term objectives with great energy, enthusiasm, and an ability to display preferable exhibitions, over what is anticipated from them, toward the accomplishment of goals. Abd Razak et al. (2010) isolated this aspect into two parts, in particular, teacher commitment to instructing work and teacher commitment to the career. The main part highlights the degree to which an individual identifies mentally with their work, and intends to participate in an effort of instruction, and the subsequent part specifies an affective connection to the job related to individual documentation and the fulfillment of functioning as an instructor.

Inside the school settings, educator experts' commitment is dictated by their feeling of inclusion during the time spent instructing, which determines the extent of exertion that they put into promoting advanced learners' education and well-being, and exceptionally committed proficient teachers are required to attain capability in new issues that add to their effort, to upgrade their capacity to manage learners' distinctive necessities, and to further develop their class execution. The high loyalty of an educator to their school shows high receptivity to class beliefs and eagerness to apply exertion in school tasks and to remain at school (Somech and Bogler, 2002). Student success is associated with a wider variety of issues containing the learner's academic capability, home atmospheres, and socioeconomic eminence that are hard to adjust to, along with school and teacher features. The main problem found was that although many studies have been done on creating an enjoyable situation in which to foster effective adjustment in schools, the impact that optimism and teacher commitment have on the learners' success is still unclear. Moreover, it is an old perception that learner success and accomplishment are indiscernible and have longstanding consequences, and it is difficult to recognize the teacher's impacts on learner accomplishment in an instantaneous way. For these reasons, the present review attempted to investigate the association of teacher commitment and academic optimism with learner educational success.

## Teacher Commitment

Teacher commitment has been regarded as a desire for work that is at the center of successful instruction. It is a requirement for great instruction (Day, 2004). It urges instructors to go about it as it is the wellspring of inspiration (Vallerand, 2008). Hence, passionate educators can create fervor in students to accomplish more. Without passion, all educational methodologies fail (Hargreaves, 1997). Subsequently, the impact of passion on student accomplishment is extensively perceived. Hansen (2001) in his endeavor to characterize a zealous educator expresses that they can urge students to be more willing and achieve more. Students accomplish more as long as they give it a second thought and are eager for learning (Fink, 2003). Teachers with commitment can give learners imaginative educational techniques that can prompt higher levels of accomplishment. Furthermore, committed educators, through urging learners to be included in school exercises, can make students energetic. Teacher commitment is vital for excellent instruction, and it incorporates a commitment to the school, learners, vocation continuation, proficient knowledge base, and instructing career (Crosswell and Elliott, 2004). Committed teachers are interested in interacting with their learners and think frequently about their growth. These instructors significantly battle for proficiency in educating and education by utilizing various methodologies. Without affection for their career, teachers cannot lead education successfully. Teachers with an undeniable degree of commitment are enamored with educating others (Linston and Garrison, 2003).

Commitment to instruction is essential to decrease educator turnover, carry out curricular advancements, authorize change inside a discipline, keep up with program progression, maintain achievement, and improve the profundity of learners' improvement (Hausman and Goldring, 2001; Ingersoll and May, 2010; Robinson and Edwards, 2012). Studies have recognized a critical scope of factors that impact commitment to instruction, featuring the requirement for research investigating the connection between different skill regions and commitment to educating others (Mee and Haverback, 2014; Sorensen and McKim, 2014; McKim and Velez, 2016).

Committed teachers might have solid mental connections to their school, their learners, or their branches of knowledge. They ought to be internally roused. Teacher commitment might be coordinated toward various substances; for instance, to the control of educating, to learners' achievement, to explicit projects, or the school as an association (Alfassi, 2004; Smith, 2010). When there are committed teachers, schools can work effectively. Consequently, establishing a positive school environment (Peterson and Skiba, 2000) can be an extraordinary assistance to assembling teachers' commitment. School managers and executives are supposed to be familiar with the components that are identified with the school environment. A constructive school culture (Ellison et al., 2005) is the basic initial step that managers need to make and maintain regardless of the difficulties of alterations. Accordingly, the school administration ought to be capable of assuming an impartial part in establishing a pleasant workplace for the educators; thereby, prompting the improvement of learners' conduct and academic accomplishments.

**Figure 1:**
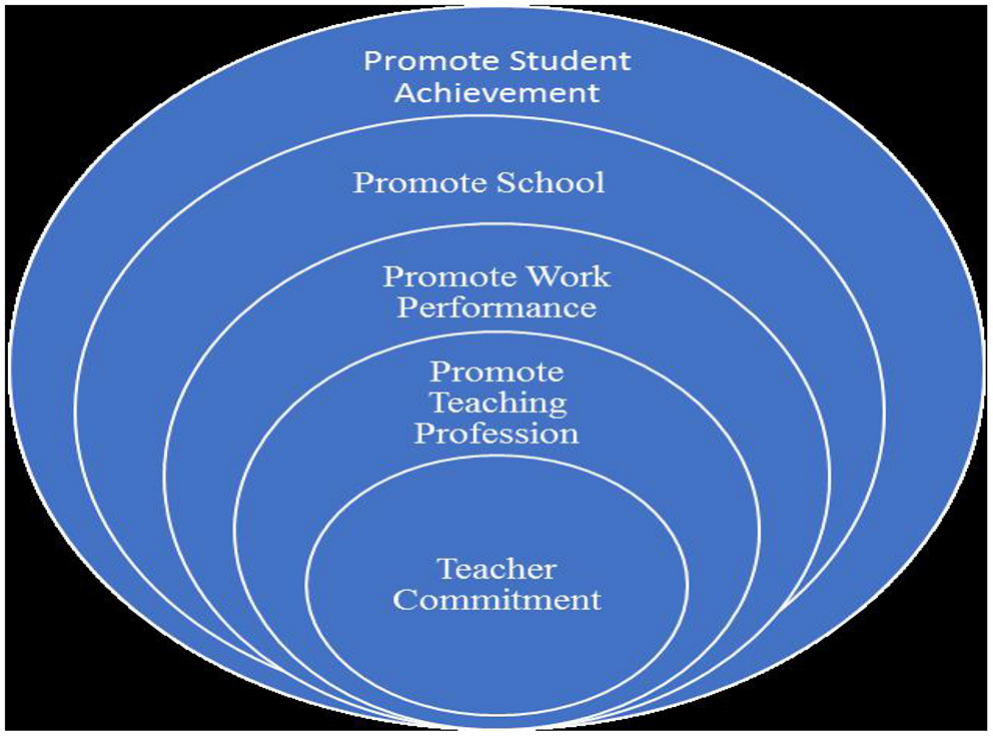
Teacher commitment.

Figure 1 demonstrates that teacher commitment is at the center of encouraging the training career, work presentation, and school and learner success. It is a fundamental part of quality schooling. Commitment gives instructors the affection, want, and energy that they need to perform better. As declared by Somech and Bogler (2002), committed teachers are believed to be happier with educating others and consistently endeavor to achieve decent teaching. They are anxious about their execution and consistently look for elevated expectations to accomplish education appropriately. Their dedication to the faculty is verifiable and their tendency toward the achievement of school objectives is obvious (Carbonneau et al., 2008). Student success needs the consideration of instructors and is influenced by teacher commitment and strong teacher-student interpersonal variables such as care, stroke, clarity, credibility, confirmation, and rapport (Xie and Derakhshan, 2021). Committed teachers consistently endeavor for greatness to have an effect on the improvement of learners (Dannetta, 2002). They take care of their qualified progress and take care of educating and learning successfully from others. It is crucial to comprehend the necessities of learners in instruction. Teacher commitment is a significant element that attracts the consideration of educators to the necessities of learners. Simultaneously, these instructors realize how to urge learners to take an interest in the learning cycle. All things considered, encouraging a dynamic participation is a way to propel learners to accomplish more.

## Teachers' Sense of Academic Optimism

Optimism addresses clear expectations for what is to come (Carver et al., 2010). Correspondingly, it is maintained that individuals with positive expectations lean toward great outcomes as much as could be expected and regularly remain objective-oriented. The present review has revealed that optimism adds to flexibility and is portrayed as the most critical component in alleviating pressure features. Also, optimism addresses people's uplifting outlook toward difficulties and is regarded as a noticeable component of resilience (Souri and Hasanirad, 2011). Utilizing Bandura's Triadic Mutual design, Hoy et al. (2006) made a system for considering educators' feeling of scholastic optimism as an administrative variable. The three components of scholarly optimism are instructors' sense of efficiency, educators' confidence in learners and guardians, and instructors' scholastic accentuation; these convictions are straightforwardly identified with student success.

As stated by Hoy et al. (2006), the term scholastic optimism was picked to address the numerous positive choices that schools make to defeat the unfortunate results of low socioeconomic status. Optimism addresses an inspirational, emotional, and psychological position toward what is to come. Specifically, optimistic people establish helpful opportunities in which beneficial things will be copious. As declared by Peterson and Seligman (2004), at any event, when confronted with a difficult undertaking, optimists endeavor to defeat obstacles to accomplish their set objectives. Thus, scholastically optimistic educators have elevated standards for all learners and individuals from their local area, continually seeing the potential for learners' education and development.

## Implications and Future Directions

The present study has some pedagogical implications for EFL teachers and teacher trainers as it supports educators and experts in the EFL teaching domain to expand their points of view on the importance of teacher commitment and optimism and its effect on the students' success. It can also promote the educators' mindfulness about their commitment. Teacher commitment is supposed to accomplish institute objectives, increase teacher competence, and nurture education. Furthermore, teacher commitment is supposed to be important in explaining the teachers' difficulties, which is not restricted to the school level; however, it happens even in the occupation that these reflections offer a requirement to underline teacher commitment systemically (Park, 2005).

The review of the literature sheds light on the requirement for EFL instructors to know about EFL optimism as a significant individual contrast and the crucial role that it can play in language education. In this manner, endeavors to make learning a foreign language a reachable objective in the perspective of learners ought to be a significant concentration in foreign language instruction and learning settings to advance learners' language education support and self-directed language learning conduct. Administrators are supposed to simplify premeditated and determined actions to build and improve trusting relations between the teaching space and the home, which in turn leads to students' success.

The present review of the literature additionally featured the massive support of L2 education optimism in language learning which has suggestions for educators and teachers as they are constantly focused on approaches of upgrading learners' education. It accentuates the necessity of concentrating on learners' optimistic perspectives toward L2 learning and offering help for learners to foster this characteristic. As a result, interventions and mediations ought to be intended to fortify and support L2 learning optimism and limit skeptical perspectives toward L2 learning.

Notwithstanding the apparent importance of a positive state of mind to language accomplishment, it appears to be that subsequent language research has been delayed in embracing an uplifting outlook. In this research, solely optimism was designated as a PP variable. Further research needs to emphasize different hypotheses related to positive disposition comprising resilience and constancy as markers of different contrasts in PP and explore their importance in language learning. A more noteworthy consideration regarding PP is following the overall pattern in scholastic works which centers on the strength-based simulations models of working on students' success (Wrosch and Scheier, 2003). Scholarly optimism can be achieved and when it happens, expanded achievement and better execution are probably going to be studied. Unmistakably, the connection between scholarly optimism and accomplishment is proportional. Optimism enables accomplishment; however, accomplishment builds up and upgrades optimism. The two notions are viable as well as reciprocal (Smith and Hoy, 2007).

The conventional perspective on accomplishment in a language setting is that achievement is an element of ability and inspiration; the skilled and inspired people are the successful ones. Seligman (2006) proposed another issue for learners' success, which is optimism, and he contends that optimism is as much as an ability or inspiration when it comes to accomplishment. Furthermore, optimism can be well-educated and created. Learned optimism is a discrete issue (Seligman, 2006) as is the scholastic optimism of educators. In fact, a significant number of the decisions about specifically educated optimism can be utilized to the scholastic optimism of educators.

Efficacy, trust, and scholastic accentuation are the crucial components in the overall concept of scholarly optimism. Four educator factors, specifically dispositional optimism, humanistic class administration, learner-focused instructing, and instructor citizenship conduct are distinguished in the improvement of educator's scholastic optimism. Scholarly optimism tries to sustain what is best in educators to improve learners' education. It is maintained that there is a genuine worth in advancing assurance, with its solidarity and resilience, instead of emphasizing disappointment, with its shortcoming, and powerlessness (Hoy et al., 2006). The essential ideas of aggregate efficacy, staff trust, and scholastic accentuation are powerfully and correspondingly identified with one another.

Educator efficacy, another noteworthy predictor of students' success, is a psychological part of scholarly optimism, the reasoning and accepting side; educator trust in learners and guardians is the emotional and passionate side of the overall concept; and educator scholastic accentuation is the social aspect, that is, the authorization of the intellectual and emotional into activities (Hoy et al., 2008). Teachers with an undeniable degree of commitment will be more faithful to faculties where they work; they will also contribute to students' achievements successfully, which is a notable impact of this paper. Commitment is laudable in light of the fact that it works with learning. In the event that great working circumstances are accommodated for committed teachers, the adequacy of the instructive association that will prompt encouraging ramifications for the school and learners will be upgraded (Mart, 2013). Teacher academic optimism may be the strength for the accomplishment of learners just as school educational optimism is regarded to be at the mutual level (Hoy et al., 2006). Correspondingly, higher levels of teacher commitment have been found to lead to an undeniable degree of better results from learners and schools. This is because committed teachers exhibit excitement toward instruction and learning, keep up with elevated requirements, set objectives for learners' execution, and advance a precise climate conducive to learning.

Further development of the components of commitment has positive results on the others and lifts optimism. For instance, further developing personnel trust in guardians and learners builds the feeling of aggregate efficacy and advances scholarly accentuation; thus, the feelings of aggregate efficacy and scholastic accentuation improve each other and reinforce staff trust (Hoy and Miskel, 2005). Optimism is a strong persuader since it centers on prospects with its solidarity, and flexibility with its associated shortcomings and powerlessness. Since it is declared that diverse educator cultural factors may have various sociocultural significances founded on their diverse cultures, individual practices, socioeconomic level, and acculturation (Clugston et al., 2000; Schwartz and Bardi, 2001), more studies should be done in order to take these issues into account which might influence the emphasis of affection, and it is an important predictor of dissimilar centers of commitment.

## Ethics Statement

The data was collected with the consent of respondent and ethical approval was taken from National University of Computer and Emerging Sciences, Islamabad, Pakistan.

## Author Contributions

DL read the relevant literature and studied EFL teachers' optimism and commitment and their contribution to students' academic success.

## Funding

This review was supported by 2020 Key projects of excellent talents support plan in universities of Anhui Province: Research on the development path of local university teachers' International Literacy (gxyqZD2020121).

## Conflict of Interest

The author declares that the research was conducted in the absence of any commercial or financial relationships that could be construed as a potential conflict of interest.

## Publisher's Note

All claims expressed in this article are solely those of the authors and do not necessarily represent those of their affiliated organizations, or those of the publisher, the editors and the reviewers. Any product that may be evaluated in this article, or claim that may be made by its manufacturer, is not guaranteed or endorsed by the publisher.
